# Ankle Fracture Surgery on a Pregnant Patient Complicated by Intraoperative Emergency Caesarian Section

**DOI:** 10.1155/2013/962794

**Published:** 2013-06-02

**Authors:** Ran Schwarzkopf, Steven C. Gross, Allen Coopersmith, Ramesh Gidumal

**Affiliations:** ^1^Department of Orthopaedic Surgery, UC Irvine Medical Center, 101 The City Drive South Pavillion III, Orange, CA 92868, USA; ^2^Department of Orthopaedic Surgery, NYU Hospital for Joint Diseases, 301 E. 17th Street, New York, NY 10003, USA; ^3^Department of Anesthesia, NYU Hospital for Joint Diseases, 301 E. 17th Street, New York, NY 10003, USA

## Abstract

We report the case of a woman in the third trimester of pregnancy who sustained an ankle fracture dislocation that could not be adequately closed reduced. After discussions with the patient, her obstetrician, and the anesthesiologists, she was indicated for surgical fixation. A heart tone monitor was used to assess fetal health during the procedure. During surgical incision, the fetus went into distress, and an emergency caesarian section was performed. After delivery of the infant and abdominal closer, surgery was completed. Due to a cohesive team effort, both the patient and her infant had excellent outcomes. There are many important considerations in the surgical management of the pregnant patient with traumatic orthopaedic injuries. Of especial importance to the orthopaedic surgeon is the impact of patient positioning on uteroplacental blood flow. This report discusses factors that should be taken into account by any orthopaedist who plans to operate on a pregnant patient.

## 1. Introduction

In the United States, traumatic injury remains a significant complication of many pregnancies. Trauma affects as many as 8% of pregnancies and represents the leading nonobstetric cause of maternal death [1–3]. While major injury can potentially result in life-threatening sequelae, minor trauma can nonetheless also create diagnostic and therapeutic challenges for the patient and surgeon. We present the case of a thirty-nine-year-old female who was thirty-six weeks pregnant when she sustained a left ankle twisting injury resulting in an ankle fracture dislocation. Her surgical procedure was complicated by fetal heart rate abnormalities resulting in emergent cesarean section. We believe this scenario, while described in the obstetrical literature, is unique in the orthopaedic literature and illustrative of a few important points related to perioperative management of the pregnant patient. Written consent for publication of this case was obtained from the patient involved.

## 2. Case Report

**Figure 1:**
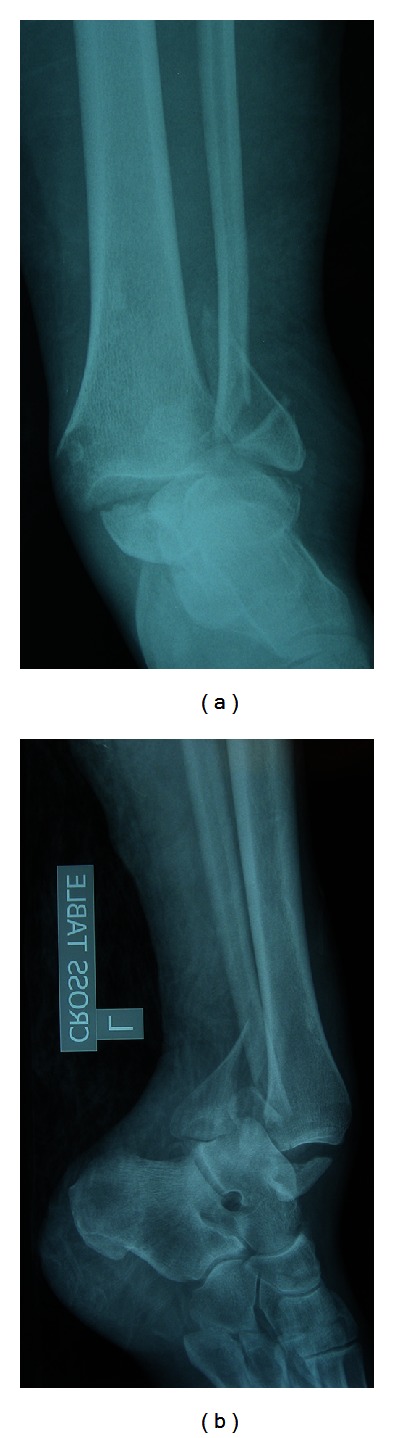
Initial anteroposterior and lateral radiographs showing an ankle fracture dislocation with lateral and posterior displacement of the talus.

A thirty-nine-year-old woman who was thirty-six weeks pregnant sustained a left ankle injury during a mechanical slip and fall, resulting in significant pain and deformity. Her pregnancy had been uncomplicated up until that point. She had no significant past medical or surgical history and did not take any medications at the time of injury. On initial presentation to the emergency department at an outside institution, the patient was diagnosed with a trimalleolar left ankle fracture dislocation (Figure 1). The fracture pattern was consistent with a Lauge-Hansen supination-external rotation IV injury. The patient's neurovascular status was intact. She was closed reduced and transferred in a splint from the outside hospital to our institution for further care.

Upon arrival at our emergency room, we reviewed the radiographic images, which revealed an incompletely reduced tibiotalar joint and proceeded to remove the splint in order to fully examine the site of injury. At this time, the ankle was noted to be highly unstable. Attempts at closed reduction and splinting yielded the same incomplete restoration of the ankle mortise with persistent subluxation of the talus. The patient was counseled that her injury was unstable, incompletely reduced, and that it would require surgical intervention. The risks and benefits of surgery, not only to the patient but also to the fetus, were discussed with her in conjunction with the obstetricians. The decision was reached to proceed the following day with open reduction and internal fixation of the left ankle.

A fetal heart rate monitor was used for continuous fetal assessment while in the operating room. Spinal anesthesia was performed with the patient in the lateral decubitus position, as this was deemed more appropriate than general anesthesia with relation to the pregnancy. Following the induction of anesthesia, the patient was placed in the supine position with a bump under her right side to displace the uterus. Sterile draping was begun. During this time, the fetal monitor began to show prolonged heart rate decelerations to 50–60 beats per minute, which did not correct with changing the patient's position. The obstetrical team, who had been previously notified about the case and was present in the hospital, was immediately called. Upon their arrival, the obstetricians decided to perform an emergent cesarean section. Though the fetal heart rate had stabilized by the time they completed their evaluation, they were committed to an emergent delivery in order to avoid further distress to the fetus.

The patient was again placed in the lateral decubitus position in order to receive an epidural catheter. After this was placed, the patient was returned supine, and a caesarian section was performed. A healthy infant female was successfully delivered, with APGAR scores of eight at one minute and nine at five minutes. The obstetricians closed the uterus and abdomen without incident.

**Figure 2:**
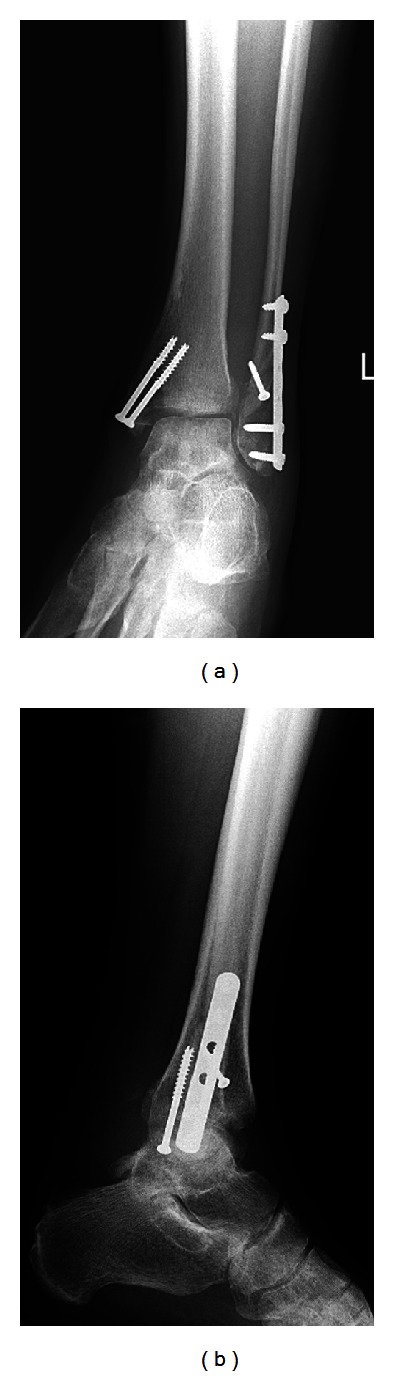
Postoperative mortise and lateral radiographs taken three months after surgery.

After the anesthesiologists and obstetricians reassessed the patient and determined that she was stable for further surgery, we proceeded with open reduction and internal fixation of the ankle (Figure 2). The patient tolerated the procedure well, and there were no further complications. Postoperatively, the patient was transferred to the postpartum unit, where she progressed well over the ensuing days in terms of rehabilitation and physical therapy. The patient was changed from a plaster splint to a fiberglass cast after several days. She was discharged from the hospital on postoperative day four in good condition. Clinical followups in the twelve months since the operation have shown no complications, with both the patient and her daughter doing well and progressing appropriately.

## 3. Discussion

Many pregnant patients in the United States undergo nonobstetric surgery each year, with current estimates placing the number at greater than 80,000 [4]. Although the vast majority of these procedures are performed without significant complication, it is important to take note of the inherent risks involved. While in many situations it is preferable to delay surgery on a pregnant patient until the postpartum period, this is not always possible. The most recent ACOG Committee Opinion on nonobstetric surgery in pregnancy stresses the importance of obtaining preoperative obstetric consultation. It also states that there are inadequate data to make specific guidelines for these procedures, including the utility of using continuous intraoperative fetal heart monitoring [5].

The current evidence for using continuous fetal heart monitoring in order to avoid adverse outcomes is anecdotal [4]. In general it is recommended that pregnant patients who have sustained traumatic injury and exhibit signs of fetal distress, including fetal heart rate of less than 100 beats per minute and/or prolonged decelerations, undergo emergent cesarean section if the fetus is older than 26 weeks [6]. However, this decision can only be made after consultation by an obstetrician. In our case, it seems that use of continuous fetal monitoring detected a significant abnormality and led to a timely intervention, potentially avoiding a poor outcome.

**Figure 3:**
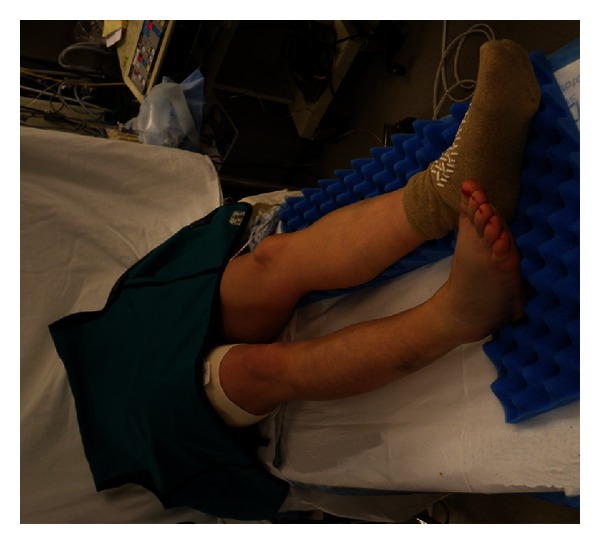
Preoperative positioning of a pregnant patient with a bump under the right hip and a lead apron draped over the abdomen and pelvis.

Surgical positioning is another important consideration in the pregnant patient, particularly in the third trimester. Placing the patient supine during this time can result in compression of the inferior vena cava by the uterus, which in turn can reduce maternal cardiac output by up to 30% and cause alterations in uteroplacental blood flow [7]. This compressive effect can be partially relieved or eliminated by angling the patient's body to the left during positioning (Figure 3). The lateral decubitus position is ideal, but the necessity of certain surgical approaches may prevent its use. In our case, the patient required a lateral incision over the left ankle as part of the planned repair, so a left lateral decubitus position was not possible. However, even though a bump was used under her right side, the fact that our patient was placed supine for the planned ankle fixation may have contributed somewhat to the onset of the observed fetal heart rate abnormalities. This highlights the importance of the orthopaedic surgeon being cognizant and flexible in terms of positioning a pregnant patient. It is recommended to avoid a purely supine position if possible. Placing a wedge under the right hip and/or tilting the operating table can help in positioning if desired.

There are some important distinctions between the goals of obstetric and nonobstetric anesthesia in the pregnant patient. Obstetric anesthesia is designed for pain relief without hindering uterine contractility. In contrast, a central function of nonobstetric surgical anesthesia is prevention of premature labor or spontaneous abortion. Also, obstetric anesthesia should avoid depression of the fetal central nervous system, whereas these concerns are not theoretically relevant in nonobstetric anesthesia [8]. For our patient, the original decision was made to use spinal anesthesia, which is associated with less fetal drug exposure, less impact on fetal heart rate variability than general anesthesia, and absent risk of unintentional dural puncture with an epidural needle [4]. However, the need for emergent cesarean section required that the patient's anesthesia be augmented with an epidural. Our patient's clinical situation emphasizes the importance of an appropriate anesthetic plan for pregnant surgical patients.

Management of pregnant patients with operative fractures is complex. A team-based approach is warranted, incorporating the orthopaedic surgeon, obstetrician, and anesthesiologist. There are many case-specific factors to consider, including patient positioning and method of anesthesia. While there is no consensus recommendation on the use of continuous intraoperative fetal heart monitoring, in our case the monitor helped identify a potential problem before it led to an adverse outcome. As a result of cohesive patient care by multiple providers in the perioperative period, we were successfully able to deliver a healthy infant as well as perform surgical fixation of an orthopaedic injury in a timely and safe fashion.

Traumatic injury complicates many pregnancies. Surgical management of orthopaedic injuries in the pregnant patient is complex and requires a team-oriented approach. Consultation by an obstetrician is mandatory preoperatively, and an appropriate plan for surgical anesthesia is essential. The use of a heart tone monitor can aid in identifying fetal distress and potentially prevent poor outcomes. The orthopaedic surgeon must be aware of limitations in anesthesia and surgical positioning when operating on a pregnant patient, particularly during the third trimester. Special attention must be given to the effect of patient positioning on uteroplacental blood flow. Through teamwork and careful planning, it is possible to deliver safe and effective surgical care to the injured pregnant patient.
